# Immune imprinting of SARS-CoV-2 responses: changing first immune impressions

**DOI:** 10.1128/msphere.00758-23

**Published:** 2024-03-13

**Authors:** J. Torresi, M. A. Edeling

**Affiliations:** 1 Department of Microbiology and Immunology, The Peter Doherty Institute for Infection and Immunity, University of Melbourne, Parkville, Victoria, Australia; Duke Human Vaccine Institute, Durham, North Carolina, USA

**Keywords:** immune imprinting, vaccine immunology, humoral immunity

## Abstract

Since the emergence of the ancestral severe acute respiratory syndrome coronavirus 2 (SARS-CoV-2) virus and the successful rollout of protective vaccines based on this original strain, SARS-CoV-2 has evolved into several variants, in a classical virus-host arms race typical of RNA viruses, to progressively evade the host immune response. Next-generation bivalent vaccines have been developed with broader protection against emerging variants than the ancestral vaccine. Nonetheless, even these vaccines show lower protection against the latest Omicron variants. Immune printing describes how an immune response to an immunogen is impacted by earlier exposures to a related immunogen. Several lessons about the effect of immune imprinting on responses to SARS-CoV-2 infection and vaccination, including age-associated impacts, can be learned from influenza. Understanding the mechanisms of imprinting of SARS-CoV-2 will be important to inform the design of vaccines that produce broader and more durable protective immune responses to emerging variants.

## PERSPECTIVE

## IMMUNE IMPRINTING AND IMPLICATIONS FOR INFECTION AND VACCINE-INDUCED IMMUNITY

Infection with respiratory viruses early in life results in an “imprinting”of immune responses such that the antigenic determinants in that or related viruses are recognized more efficiently in future exposures. The result is that humans maintain a lifelong reactivity against viruses they are exposed to in childhood, and this may be beneficial in producing rapid immune responses against viral pathogens. However, immune printing incorporates both negative and positive effects of the primary immune response on subsequent immune responses to a distantly related antigen exposure, be it through infection or vaccination. As such, antibody responses may become biased toward viruses an individual is exposed to in childhood and may interfere with the development of protective responses against new viral threats.

Imprinting of antibody responses has been well described for influenza infection and vaccination (1) and has also been increasingly described for SARS-CoV-2 infection (2–4). However, our understanding of the effects of immune imprinting on the responses to SARS-CoV-2 vaccines and/or new variants remains unclear, yet understanding how imprinting might affect the immune response of COVID-19 vaccines will have important implications for future vaccine approaches as emerging variants shift further antigenically from current circulating viruses. Several lessons about the effect of immune imprinting on responses to both infection and vaccination can be learned from several decades of experience with influenza.

## INFLUENZA AND IMMUNE IMPRINTING

Antibodies against influenza will bind strongly to a viral strain against which these antibodies were initially elicited. However, they may bind poorly to variants that have drifted from the founding strain (5, 6). What has become apparent is that antigens from strains of influenza viruses encountered in childhood are given a “senior” position in a hierarchy of immune responses, and subsequent exposure to the same or closely related subtypes will boost immunity to these previously encountered strains. In contrast, influenza viruses encountered later in life assume progressively “less senior” positions in the immune hierarchy (7), and this may impact on the development of effective immune responses against later strains such that antibody responses acquired later in life may provide less protection than childhood imprinted responses (8).

Several studies provide evidence of the effect of pre-existing immune memory and antibodies on subsequent viral exposure. In a study investigating the importance of immune imprinting, investigators looked at the severity of influenza by birth cohorts (cohorts based on initial childhood exposure to an influenza subtype rather than age groups) during the 2009 pH1N1 pandemic and found a significant difference in hospitalization and mortality according to the year of birth and the predominant circulating influenza subtype (H3 vs H1) (9). Prior to 2009, the number of hospitalizations and deaths due to influenza was higher in H3 compared with H1 predominant seasons in all birth cohorts. In contrast, after the emergence of 2009 pH1N1, H1 predominant seasons produced more severe disease and a greater number of deaths in birth cohorts born after 1957, while birth cohorts born before 1947 continued to have more deaths in H3 seasons (9). A correlation between susceptibility to severe influenza and initial influenza exposure, or birth cohort, was similarly seen in Mexico, where the 2009 pH1N1 pandemic revealed a disproportionately higher number of cases of severe pneumonia and deaths in individuals aged 5–59 years compared to individuals over the age of 60 years. Overall, 87% of deaths and 71% of cases of severe pneumonia occurred in individuals aged 5–59 years compared to 17% and 32%, respectively, in previous influenza seasons that were predominated by influenza subtypes other than H1N1. This represented up to an 11.7% increase relative to previous referent periods compared to a 0.3% increase in individuals over the age of 60 years (10). The higher morbidity and mortality in the 5- to 59-year-old cohorts were believed to reflect the preferential development of immune responses to influenza subtypes other than 2009 pH1N1. In further support of a link between birth cohort and disease susceptibility, in a serological study using sera collected during the 2009 pH1N1 pandemic, the prevalence of pre-existing cross-reactive neutralizing antibody to 2009 pH1N1 was found to be highest in individuals born before 1950 (34%) compared to individuals born after 1980 (4%) (11). Finally, that H5N1 and H7N9 cases were highest in the young and old, respectively, was explained by childhood imprinting (12). Specifically, a modeling study that examined influenza A virus (IAV) exposure according to year of birth demonstrated that an individual’s first IAV (H1N1) infection in childhood conferred lifelong protection against severe disease from novel HA subtypes (H3N2) in the same phylogenetic group (12). The broadly protective responses to the first H1N1 infection persisted despite decades of natural exposure to H3N2 (12). In summary, protection against influenza may be determined by the exposure to influenza viruses circulating around the time of an individual’s year of birth.

## INFLUENZA VACCINES AND A POTENTIAL NEGATIVE EFFECT OF IMMUNE IMPRINTING

**Fig 1:**
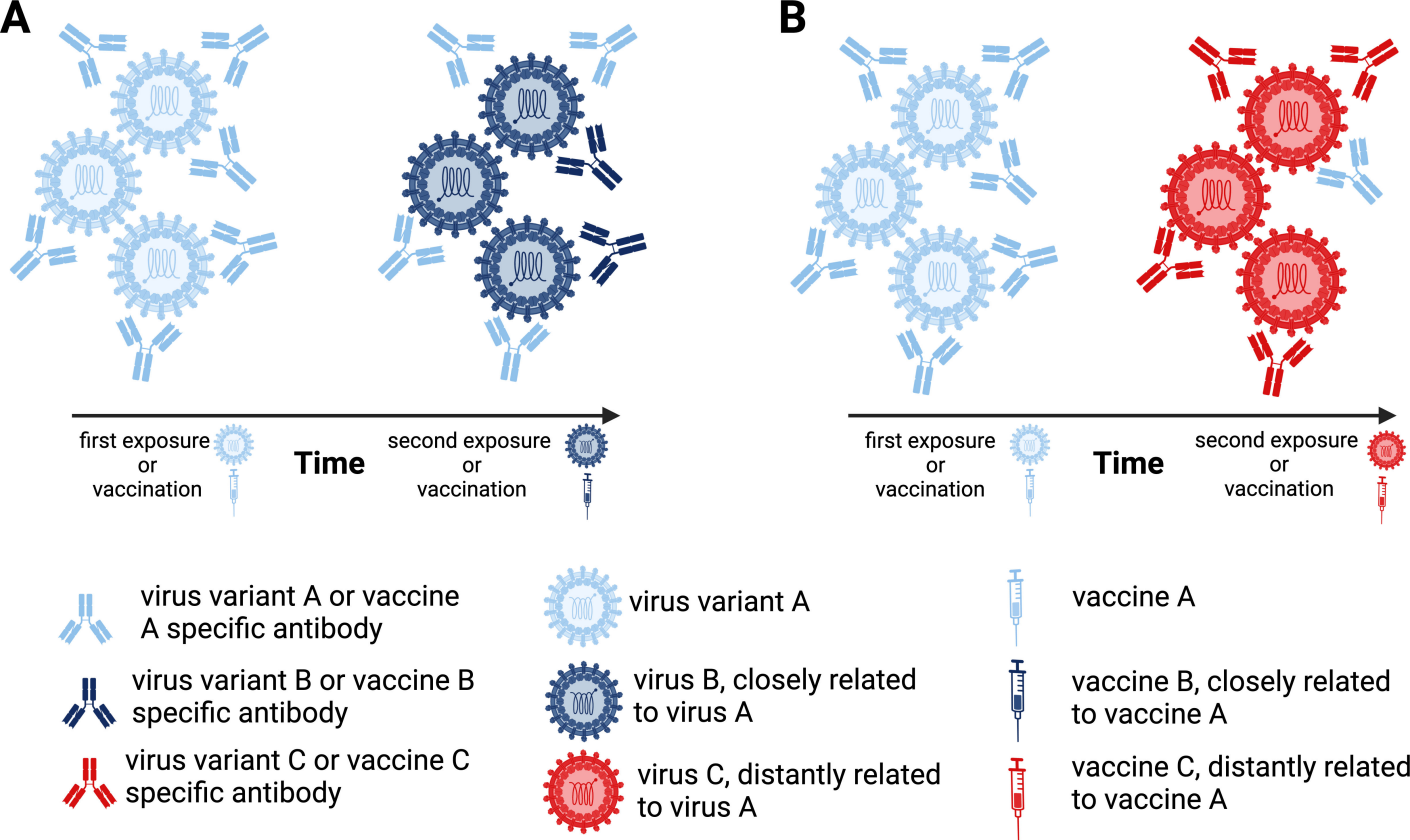
Potential changes in the antibody landscape due to a potential impact of immune imprinting by viral exposure through infection or vaccination. (**A**) A first exposure to virus A (light blue) (**A**) alters antibody responses to a subsequent infection with a closely related virus, virus B (dark blue), so that the prevailing antibody response in the second infection is predominantly directed to virus A. (**B**) A first exposure to virus A (light blue) followed by an exposure to an antigenically distant virus, virus C (red), results in the predominant production of high-affinity neutralizing antibody to the distant virus while also stimulating low-titer antibodies to the earlier infecting virus, virus A. The imprinted response to viral infection also applies to vaccination. Repeated vaccination with the same vaccine produces antibody responses that are predominantly directed to the original vaccine (left panels in A and B). Subsequent immunization with a vaccine containing a closely related antigen results in the predominant production of antibody to the original vaccine and a low-titer antibody to infection with a closely related virus B (right panel in A). In contrast, immunization with an antigenically distant vaccine is less influenced by prior imprinted antibody responses, resulting in the production of high-titer neutralizing antibody to virus C and lower-titer antibody responses to earlier infecting virus A (right panel in B). Created with BioRender.com.

Just as imprinting by exposure to pandemic strains of influenza early in life may increase susceptibility to antigenically distant strains during future influenza pandemics (5), so too can influenza vaccination. The efficacy of influenza vaccines from year to year is impacted by the antigenic relatedness of past vaccines, the strains represented in current vaccines, and circulating epidemic variants. Vaccine efficacy can be predicted to be high when the antigenic distance between all three strains is close but low if the antigenic relatedness between the vaccine and epidemic strains is distant, indicating that vaccine antigen selection needs to be carefully considered (5) (Fig. 1).

The match of a virus to a vaccine’s antigenic specificity may not only be affected by antigenic distance between viral subtypes. During the 2018/2019 H3N2 epidemic in Canada, the odds of a medically attended H3N2 3C.3a influenza illness were more than fourfold higher in a 1964–1983 birth cohort than in a matched unvaccinated cohort (13). The vaccinated cohort had been vaccinated in the preceding 2 years with H3N2 clade 3C.2a and revealed significantly lower vaccine efficacy compared to other vaccinated birth cohorts, despite H3N2 clade 3C.3a imprinting in and after the H3N2 1968 epidemic. The separation between the two H3N2 clades consisted of a shielding glycosylation at a critical amino acid in the HA head, not present in the contemporary 3C.3a virus but present in the vaccine 3C.2a HA (13). A similar effect was later reported in Europe during the 2018/2019 H3N2 epidemic (14). Again, the low vaccine efficacy in this age group most likely resulted from genetically distinct differences between the vaccine seed virus and the circulating clade 3C.3a virus.

## OVERCOMING INFLUENZA IMMUNE IMPRINTING

An important question to address is whether the negative effects of immune imprinting can be overcome by later exposure to different viral subtypes. On the one hand, the hierarchy of immune response can be reset in individuals with pre-existing imprinted responses when exposure to a new pandemic virus occurs early in teenage or adult life, particularly before the age of 20 years (15). Several studies have also shown that repeated influenza vaccination maintains a high level of protection against severe influenza, arguing that potential imprinting from a prior vaccine exposure may not impact negatively on seasonal vaccine efficacy (16–19). On the other hand, vaccination with seasonal vaccine can be associated with a progressive loss of vaccine efficacy especially in individuals over the age of 65 years and with increasing numbers of previous vaccinations (20–22). Furthermore, vaccine efficacy in a current season also remains highest in individuals who have not been repeatedly vaccinated in previous seasons (21). The relationship between repeated vaccination and imprinting requires further investigation.

The effect of imprinting can also be overcome by including adjuvants with vaccines. In a mouse model of influenza infection, the use of dendritic cell (DC)-stimulating adjuvants such as CpG ODN or a squalene-based oil-in-water adjuvant in both secondary and primary immunization induced stronger antibody responses to the second virus. Sequential infection in the absence of adjuvants failed to overcome the effects of imprinting, resulting in significantly lower Nab titers to a second virus. These adjuvants shift antigen presentation away from memory B cells and to DC that enhance CD4+ and CD8+ T-cell responses and recruit naïve B cells (23). The breadth of cross-reactive antibody and the production of protective antibody against heterologous virus are also improved by the addition of squalene-based oil-in-water adjuvants (23). The effect of imprinting can also be overcome by repeatedly immunizing with a second, more distantly related viral antigen. By sequentially immunizing mice with DNA vaccines or whole inactivated virus, boosting with virus or antigen of the second virus 28 days later, at a time when memory B cells have developed, achieves significantly higher titers of protective antibodies to the second virus (23).

## COVID-19 AND IMMUNE IMPRINTING

Similar factors to influenza infection and vaccines may also apply to current COVID-19 vaccines and emerging variants like Omicron BA.1, BA.2, BA.4/5, BQ.1.1, XBB.1.5, and EG.5. A potential bias in immune response toward an earlier infecting variant or an ancestral COVID-19 vaccine could interfere or, alternatively, complement an immune response to variant-specific vaccines, depending on antigenic relatedness.

Although infection with viral variants produces variant-specific antibody responses, prior vaccination with WuH-1 S containing COVID-19 mRNA vaccines has been shown to imprint antibody responses toward the ancestral virus rather than to variant antigens (4). So prior mRNA vaccination with a WuH-1 vaccine followed by Alpha or Delta infection results in stronger antibody response toward WuH-1 virus and decreased antibody responses to viral variant epitopes compared to unvaccinated individuals infected with these variant viruses. In contrast, individuals infected with Alpha or Delta variants and with no history of vaccination develop antibodies with stronger binding to Alpha or Delta variant receptor binding domain (RBDs) compared to WuH-1 RBD (3).

Conversely, COVID mRNA vaccinees who have been previously infected with SARS-CoV-2 (hybrid immunity) are protected more from infection and severe SARS-CoV-2 disease than infection-naïve vaccinees (24, 25). The hybrid cohort is characterized by more robust and sustained antibody and B-cell responses than the vaccinated but infection-naïve cohort (26). A third dose is required to rescue the humoral (but not the CD4+ T-cell cytokine profile) response in the infection-naïve vaccine cohort to similar levels as the hybrid cohort (26).

## A SECOND CHANCE TO CHANGE FIRST IMMUNE IMPRESSIONS TO COVID-19

Bivalent vaccines that incorporate the ancestral WuH-1 strain, together with a variant strain, partially overcome the effects of imprinting from monovalent ancestral vaccines. So bivalent vaccines that incorporate either Beta (mRNA-1273.211) (27), Omicron BA.1 (mRNA-1273.214) (28), or BA.4/BA.5 (BNT162b2) (29) all produce greater Nab responses against variants than a monovalent ancestral vaccine while maintaining responses against the ancestral virus. Therefore, an antigenically divergent vaccine can produce strong variant-specific responses while recalling an ancestral response in individuals with or without a history of COVID-19 infection (Fig. 1). However, the activity of more recent bivalent vaccines against latter variants, including BA.2.75.2, BQ.1.1, and XBB.1 variants, remains lower than those against other Omicron variants, arguing that improved variant vaccines are still required (30). Furthermore, careful consideration needs to be given to the selection of variant antigens for future vaccines. For example, in dual vaccinated individuals, a third antigen exposure through infection with Delta SARS-CoV-2 increases the breadth of memory B-cell and cross-reactive Nab responses (31). In contrast, infection with Omicron BA.1 increases strain-specific memory B cells but not the breadth of immunity compared to a third dose of a mRNA vaccine, highlighting that not all variant antigens are necessarily equal in producing broadly cross-reactive Nab (31).

The optimization of future COVID-19 vaccines will benefit from a greater understanding of the role of pre-existing antibodies in the response to emerging COVID-19 variants. To this end, peripheral (pre-existing) antibodies have recently been shown to either enhance or inhibit recruitment of naïve B cells into germinal centers for the development of variant-specific responses: broad, low-affinity antibodies enhance, but high-titer, high-affinity, epitope-focused antibodies inhibit cognate naïve B cells on secondary challenge (32). In another recent finding, a boost in Omicron neutralization after a third, but not a second, dose of ancestral mRNA vaccine results from pre-existing antibodies that mask immunodominant epitopes that are mutated in Omicron to promote B-cell responses against subdominant epitopes more conserved in Omicron (33).

## CONCLUSION

The effects of imprinting of ancestral SARS-CoV-2 vaccines and infections on immunity to SARS-CoV-2 variants are rapidly emerging. Despite waning protection from infection, SARS-CoV-2 vaccine efficacy remains robust against severe disease (34). Understanding the mechanisms of imprinting of SARS-CoV-2, as has been established for influenza, will be important to inform the design of vaccines that produce broader and more durable protective immune responses to emerging variants.
